# Functional cure for chronic hepatitis B: accessibility, durability, and prognosis

**DOI:** 10.1186/s12985-021-01589-x

**Published:** 2021-06-03

**Authors:** Aixin Song, Xiao Lin, Xinyue Chen

**Affiliations:** First Department of Liver Disease Center, Beijing Youan Hospital, Capital Medical University, Beijing, China

**Keywords:** Hepatitis B virus, HBsAg clearance, Prognosis

## Abstract

Hepatitis B surface antigen (HBsAg) clearance is regarded as the ideal endpoint for antiviral treatment in terms of drug withdrawal safety and improvements in prognosis. However, the overall rate of HBsAg clearance is low and differs based on treatment method and course. The recent application of combined and extended treatment strategies have improved the HBsAg clearance rate, and several patients achieved HBsAg clearance in clinical treatment. In addition, the durability of and clinical outcomes after HBsAg clearance have become the focus of both researchers and clinicians. This article reviews HBsAg clearance in terms of accessibility, durability, improvements in prognosis and relevant advances.

## Introduction

Chronic hepatitis B virus (HBV) infection is a severe public health problem, and it is a leading cause of cirrhosis and hepatocellular carcinoma (HCC) [1, 2]. With the development of antiviral therapies in the past decade, the treatment goal for chronic hepatitis B (CHB) has been elevated beyond viral suppression (i.e., sustained undetectable HBV DNA levels) and seroconversion/loss of hepatitis B e antigen (HBeAg). HBV guidelines proposed hepatitis B surface antigen (HBsAg) clearance with or without anti-HBs as the standard clinical cure and the ideal endpoint for antiviral treatment, which should be pursued as far as possible in suitable CHB patients [3–6]. Several patients, including CHB patients, inactive HBV carriers (IHCs) and patients with cirrhosis, have achieved this clinical treatment goal. However, there are few reports on the accessibility, durability and long-term prognosis of this therapy endpoint. Therefore, the present review focuses on these points of HBsAg clearance.

## Accessibility and HBsAg clearance rate

HBsAg clearance occurs spontaneously or via antiviral treatment in CHB patients. The most commonly used drugs are nucleos(t)ide analogue (NA) and pegylated interferon (Peg-IFN). NA drugs include entecavir (ETV), tenofovir disoproxil fumarate (TDF) and tenofovir alafenamide fumarate (TAF). The 2018 AASLD guidelines recommend Peg-IFN, ETV, or TDF as the preferred initial therapy for adults with immune-active CHB. It also suggests that alanine transaminase (ALT) levels be tested at least every 6 months for adults with immune-tolerant CHB to monitor for potential transition to immune-active or immune-inactive CHB [7]. The 2017 EASL guideline recommends ETV, TDF and TAF as the preferred monotherapy regimens, and the extension of the duration of Peg-IFN therapy beyond week 48 may be beneficial in selected HBeAg-negative CHB patients [4]. The potential side effects of NAs include lactic acidosis for ETV and nephropathy, osteomalacia, lactic acidosis for TDF. CHB patients should be clinically monitored. The most frequently reported side effects for Peg-IFN are flu-like syndrome, myalgia, fatigue, mood disturbances, weight loss, hair loss and local reactions at the site of injection, and these side effects may be partially managed with dose reduction [7]. Currently, the clearance of HBsAg is based primarily on sequential or combined treatment with NA and Peg-IFN.

### Spontaneous clearance

Two large meta-analyses reported spontaneous clearance of HBsAg in 2019. Zhou et al. [8] analysed 56 studies, including 39 prospective studies and 17 retrospective studies. Thirty studies were performed in Asian populations, which accounted for a majority of the included studies. Eighteen studies were from Europe, 7 studies were from the Americas, and 1 study was from Australia. A total of 48,972 CHB patients were included. The cumulative number of follow-ups was 352,381 person-years, and 3837 patients obtained spontaneous HBsAg clearance, which resulted in a pooled annual clearance rate of 1.17%. There were differences in clearance rates between studies according to different patient characteristics. The annual clearance rate was twice as high in baseline HBeAg-negative patients (1.44%) than HBeAg-positive patients (0.74%), and it was 1.24% in IHCs (HBV DNA < 2000 IU/mL with normal ALT). Studies with a mean cohort age of 40 years and older had a higher rate of HBsAg clearance than studies with cohorts younger than 40 years and corresponded to an annual rate of 1.7% in cohorts older than 40 years versus 1.1% in the younger cohorts. Patients with cirrhosis at baseline had an annual rate of 0.8–1.4%, and there was no significant difference from the overall patients. There was no difference in the rate between different regions. Yeo et al. [9] analysed 34 cohort studies. The study sample sizes ranged from 217 to 6621 patients, and a total of 42,588 CHB patients were enrolled, including a small number of patients who received antiviral therapy. The results showed that the annual rate of HBsAg clearance in untreated patients was 1.31%, which was consistent with the Zhou et al. meta-analysis. These two analyses found that patients who were HBeAg negative at baseline and had lower quantitative HBsAg experienced higher rates of HBsAg clearance [8, 9]. Lower baseline serum HBsAg levels may predict HBsAg clearance, especially when the HBsAg levels are less than 100 IU/mL [10], which is consistent with the prediction of HBsAg clearance after antiviral therapy.

### HBsAg clearance after NA treatment

There are few large or conclusive studies on the clearance of HBsAg after NA treatment, and some of these studies are single-centre retrospective studies. Kim et al. [11] reported a clearance rate of 1% or less in 110 CHB patients who were treated with ETV/LAM (lamivudine) for approximately 1 year. A retrospective study by Yip et al. [12] reported an HBsAg clearance rate of 2.1% after an average follow-up of 4.8 years in 20,263 CHB patients treated with ETV/TDF for longer than 6 months. Wong et al. [13] retrospectively evaluated 1072 CHB patients on antiviral therapy (ETV/TDF) for approximately 6 years and found an HBsAg clearance rate of 4.58%. This study found no significant difference in the clearance rate between HBeAg-positive and HBeAg-negative patients, but the rate in patients with cirrhosis (1.15%) was significantly lower than patients without cirrhosis (4.89%). These results suggested that the clearance rate of non-cirrhosis patients was higher after NA treatment, which is not consistent with the results of patients who experienced spontaneous clearance. Compared to patients with normal baseline ALT, patients with higher ALT levels had significantly higher rates of achieving HBsAg clearance. In general, the clearance rate may increase with the extension of treatment in CHB patients, but the overall rate with currently available NA treatment is low. The HBsAg clearance rates were 1.4–5.1% after an average follow-up of 2–7 years after NA treatment [11–16].

### HBsAg clearance after Peg-IFN treatment

Various studies showed that the clearance rate of HBsAg ranged from 2.1 to 20% in patients treated with Peg-IFN [17–19]. Most of these studies were prospective or randomized controlled trial (RCT) cohorts, and the overall clearance rate was higher than spontaneous clearance and NA monotherapy. Yang et al. [20] enrolled 144 HBV patients in a prospective RCT. These patients randomly received ETV monotherapy (n = 70) or Peg-IFN add-on therapy from weeks 26 to 52 (n = 74). Patients were followed-up for at least 2 years. The results showed that the HBsAg conversion rate was 1.8% in the ETV monotherapy group and 4.1% in the latter group. Bourliere et al. [21] also performed a randomized and open-label trial in 2018. They randomly allocated 185 HBeAg-negative CHB patients: 92 patients received a 48-week course of Peg-IFN in addition to the NA regimen, and 93 patients continued to receive NAs only. HBsAg loss was reported in 3 of 93 (3.2%) patients in the NA-only group versus 7 of 90 patients (7.8%) in the Peg-IFN plus NA group at week 96. Marcellin et al. [22] also performed an RCT. This study randomly assigned 740 CHB patients to receive TDF plus Peg-IFN for 48 weeks (group A), TDF plus Peg-IFN for 16 weeks followed by TDF for 32 weeks (group B), TDF for 120 weeks (group C), or Peg-IFN for 48 weeks (group D). At week 72, 9.1% of subjects in group A had HBsAg clearance compared to 2.8% of subjects in group B, none of the subjects in group C, and 2.8% of subjects in group D. These results showed that HBsAg clearance only occurred with Peg-IFN treatment, and the clearance rate was highest in patients who received TDF plus Peg-IFN for 48 weeks. The follow-up data at week 120 [23] showed that the rates of HBsAg clearance at week 120 were significantly higher in group A (10.4%) than group B (3.5%), group C (0%), and group D (3.5%). In addition, Peg-IFN treatment may lead to a higher clearance rate for some special populations, such as CHB-infected postpartum women. Lu et al. [24] enrolled 68 CHB pregnant women with normal levels of ALT and high levels of HBV DNA who were treated with telbivudine (LDT) during the third trimester of their pregnancy. Thirty (30/68) of these women had increased ALT levels ≥ 2 × ULN with an HBeAg titre decrease > 20% or HBV DNA decrease > 2 log_10_ after delivery. These patients were treated with ADV plus Peg-IFN for 96 weeks. They found that 26.7% (8/30) achieved HBsAg clearance.

These controlled studies indicate that extended treatment, combined treatment with NA and Peg-IFN or extended treatment with combined treatment improved the rate of HBsAg clearance. Due to the different characteristics of patients and the different strategies and courses of antiviral treatment in these studies, the clearance rates were different to some extent. Therefore, on the basis of previous studies, we proposed that Peg-IFN treatment should be more individualized and should not be limited to a fixed course. Prolonged treatment with a fixed target and indefinite course of treatment may improve treatment effectiveness. In addition, there was another study of a special population. Cao et al. [25] evaluated the feasibility of Peg-IFN as a therapeutic option for inactive HBsAg carriers (IHCs, HBsAg < 1000 IU/mL and HBV DNA < 2000 IU/mL). There were 144 IHCs enrolled and divided into a therapeutic group with Peg-IFN (n = 102) and a control group (n = 42). The results showed that the HBsAg clearance rate and seroconversion rate in the treatment group were 29.8% and 20.2% at week 48, respectively, and increased to 44.7% and 38.3% at week 96, respectively. These rates were higher than CHB patients. They also found that 59.6% of the subjects showed an elevated ALT level at week 12 in the Peg-IFN treatment group, and week 12 ALT elevation was a strong predictor for HBsAg clearance at week 96. IHCs had previously been defined as dynamically observable without treatment, but this study indicated that IHCs were treatable, highly responsive to treatment, and an "advantageous population" that was capable of attaining HBsAg clearance. Extended and combined treatment actually led to better effectiveness in this population. A retrospective analysis [26] showed that low levels of baseline HBsAg and the HBsAg level 48 weeks after treatment were favourable predictors of HBsAg clearance in IHCs. Therefore, the level of HBsAg may be continuously reduced in NA-based treatment regimens combined with Peg-IFN in stages for patients with low HBsAg levels, and the overall HBsAg clearance rate may be ultimately improved [27]. However, these studies had relatively few patients, and we must be cautious when interpreting these data.

## Durability and related factors after HBsAg clearance

When patients with HBeAg-positive CHB achieve a satisfactory antiviral treatment endpoint (e.g., HBeAg seroconversion), the clinical recurrence is 20–40%, and the virological recurrence can be as high as 80–90% after drug withdrawal [28, 29]. Because the safety of drug withdrawal is uncertain, HBsAg clearance is recommended as the ideal treatment endpoint for CHB patients. The accessibility and rate of HBsAg clearance was mentioned above, but the durability of HBsAg clearance after treatment cessation remains controversial.

There is no unified definition of recurrence after HBsAg clearance or conversion, and most studies defined recurrence as the reappearance of HBsAg or HBV DNA. One study recently [30] reported that the recurrence type was defined as the reappearance of HBsAg, HBV DNA, or both during follow-up after treatment cessation. Wu et al. [30] investigated 238 CHB patients who achieved HBsAg clearance after Peg-IFN treatment. Eighteen recurrence cases were observed during a median 160-week follow-up time after treatment cessation. The cumulative recurrence rates were 0.84%, 6.29%, 6.88%, 8.18%, and 9.66% at 26 weeks, 52 weeks, 78 weeks, 104 weeks, and 597 weeks, respectively. Patients with HBV S-region variation and drug resistance had a higher recurrence rate, and high levels of anti-HBs were a protective factor against recurrence. High-risk populations for recurrence should receive follow-up and monitoring after treatment discontinuation. Li et al. [31] also performed a study with 176 HBeAg-negative CHB patients with HBsAg clearance after IFN/Peg-IFN treatment. They found that the cumulative rates of HBsAg reversion and HBV DNA reversion were 12.79% and 2.33%, respectively, after treatment cessation at a 48-week follow-up. Patients with consolidation treatment ≥ 12 weeks and high anti-HBs levels were associated with a significantly higher rate of sustained functional cure. Kim et al. [11] included 110 CHB patients who achieved HBsAg clearance after NA treatment. The recurrence rates were 7.6% and 11.9% at 12 months and 36 months, respectively. Alawad et al. [32] included 65 patients who achieved HBsAg clearance spontaneously or after NA/Peg-IFN treatment and found that 3 patients had HBsAg reversion after a 115-month follow-up. These patients all received NA or Peg-IFN treatment, and the recurrence rate was 7% in post-treated patients. The overall cumulative recurrence rates were 1.6% and 5.4% at 1 year and 5 years, respectively. The recurrence rate after HBsAg clearance varied from 1.7 to 23.8% in these and other related studies because of the different regions, characteristics and follow-up times of patients and the different clearance methods. There was no significant difference in the recurrence rate between different HBsAg clearance methods [33–36]. Most studies showed that HBsAg clearance was durable and safe during long-term follow-up after treatment discontinuation, and it was the optimal treatment endpoint for CHB patients. However, HBsAg clearance does not indicate virus eradication [37, 38]. Due to the presence of cccDNA, HBV reactivation is possible. Several studies provided explanations for cases of recurrence, including patients who received immunosuppressive or hormone therapy and the detection of drug resistance sites during NA treatment before HBsAg seroclearance [30, 39, 40]. Patients with S region mutations may develop an intracellular retention of HBsAg proteins [41]. These conditions increase the risk of recurrence in patients with HBsAg seroclearance and require surveillance.

HBeAg status should also receive attention in the pursuit of HBsAg clearance. The clearance of HBsAg in most patients is based on HBV DNA suppression and HBeAg seroconversion, but a few patients exhibit different HBsAg response patterns, such as HBsAg clearance without HBeAg seroconversion. Only HBsAg clearance based on HBV DNA suppression and HBeAg seroconversion is safe for drug withdrawal [42].

## Prognosis improvement after HBsAg clearance

HBsAg clearance is the ideal endpoint for antiviral treatment, but the ultimate goal for CHB patients is to reduce the risk of HCC and improve prognosis. Whether the ideal endpoint is associated with significantly favourable clinical outcomes has become a clinical focus. Liu et al. [43] performed a meta-analysis of 28 studies with 34,952 patients who achieved HBsAg clearance, and the follow-up time ranged from 1.6 to 15.1 years after clearance. They found that the pooled proportion of HCC development after HBsAg seroclearance was 2.29%. Eight of 28 studies compared the incidences of HCC in HBsAg clearance patients to those in HBsAg-positive patients. The results showed that 1.87% (15/803) of patients developed HCC after HBsAg clearance, and 6.40% (278/4346) of HBsAg-positive patients had HCC during follow-up. The risk for HCC in patients who achieved HBsAg clearance was significantly lower than that in patients who were persistently HBsAg positive. Several studies showed that the incidence of HCC after HBsAg clearance ranged from 0% to 4.8% in CHB patients without coinfection [12, 44–47], and the 5-year risk of HCC in HBsAg-positive patients was 18.8% and 6–7.7% in patients without HBV DNA inhibition [48] and patients who achieved HBV DNA suppression and HBeAg seroconversion [48, 49], respectively. The incidence of HCC also significantly increases in patients with cirrhosis. Lee et al. [50] found that serum HBsAg levels were associated with cirrhosis and HCC in HBV patients. Their study showed that the cumulative risk for cirrhosis was 4.8%, 8.8%, and 16.2% and that the cumulative risk for HCC was 1.4%, 4.5%, and 9.2% for patients with serum HBsAg levels < 100 IU/ml, 100–999 IU/ml, and ≥ 1000 IU/mL, respectively. Patients without baseline cirrhosis were associated with a significantly lower HCC incidence after HBsAg clearance [36]. Liu et al. [43] reported that the pooled incidence of HCC decreased further from 2.29 to 1.55% when patients with cirrhosis and coinfection were excluded. Cirrhosis is a crucial risk factor for HCC occurrence, and the early acquisition of HBsAg clearance using treatment leads to favourable clinical outcomes.

These related studies provide clear recommendations that patients who achieve HBsAg clearance have favourable clinical outcomes compared to patients who achieve only HBV DNA suppression and HBeAg seroconversion. HBsAg clearance leads to biochemical, virological and liver histological improvements, and it significantly reduces the risk of HCC. However, HCC may occur after HBsAg seroclearance despite it being the ultimate treatment endpoint recommended by current guidelines. The risk factors associated with HCC include the presence of cirrhosis, male sex, and age ≥ 50 years at the time of HBsAg clearance [51]. Closer attention should be given to patients with one or more of these risk factors.

These high-risk patients should be re-examined in a timely manner even if HBsAg clearance is obtained. These results also suggest that achieving a functional cure early in the absence of cirrhosis results in a better prognosis [52, 53].

## Conclusions

In conclusion, HBsAg clearance may be pursued as the ideal treatment goal. Combined treatment with NA and Peg-IFN or Peg-IFN add-on therapy and prolonged treatment lead to a higher rate of HBsAg clearance, especially in special populations, such as postpartum patients and IHCs. The drug may be safely withdrawn by consolidation therapy after HBsAg clearance, and clearance is sustainable, with an overall recurrence rate of approximately 10%. Consolidation treatment and high anti-HBs levels are protective factors against recurrence, but patients with a history of drug resistance and S-region variation must be constantly monitored. Patients who experience HBsAg clearance have a significantly decreased HCC incidence and favourable clinical prognosis, and patients without baseline cirrhosis have a significantly lower HCC incidence and better long-term prognosis than patients with cirrhosis. Therefore, it is feasible to pursue HBsAg clearance, and we emphasize the importance of obtaining HBsAg seroclearance early.

Notably, most of the infected individuals had a fairly low chance of a cure with the currently available antiviral drugs. However, novel therapies aimed at functional cure are under active development, including HBV core protein inhibitors, drugs targeting cccDNA, siRNAs targeting viral transcripts, and immune modulators with different modes of action [37]. These drugs and therapies may play a vital role in suppressing HBV DNA and HBV RNA, producing a meaningful decline in HBsAg levels, and restoring HBV-specific immune responses [54–56]. The development of combination strategies towards a functional cure would dramatically reduce the disease burden associated with CHB infection. Novel drugs and therapies of innovative molecules will ultimately lead to the functional cure of CHB in the near future.

## Data Availability

Not applicable.

## Abbreviations

HBV: Hepatitis B virus
HBsAg: Hepatitis B surface antigen
CHB: Chronic hepatitis B
HBeAg: Hepatitis B e antigen
HCC: Hepatocellular carcinoma
IFN: Interferon
PEG-IFN: Pegylated interferon
NAs: Nucleos(t)ide analogue
anti-HBs: Hepatitis B surface antibodies
IHCs: Inactive HBV carriers
ETV: Entecavir
TDF: Tenofovir disoproxil fumarate
TAF: Tenofovir alafenamide fumarate
LAM: Lamivudine
